# Risk prediction models for peristomal moisture-associated skin damage in China: a systematic review and meta-analysis

**DOI:** 10.3389/fmed.2026.1789674

**Published:** 2026-06-04

**Authors:** Haijia Liu, Mengzhen Huang, Yuanfan Yang, Fen Zhao, Huilin Long, Bo Huang

**Affiliations:** The Second Affiliated Hospital, Jiangxi Medical College, Nanchang University, Nanchang, China

**Keywords:** evidence-based nursing, meta-analysis, peristomal moisture-associated skin damage, risk prediction model, systematic review

## Abstract

**Background:**

Peristomal Moisture-associated Skin Damage (PMASD) is a complication of enterostomy that significantly increases the healthcare burden and contributes to poorer patient prognoses. Risk prediction models for PMASD offer significant utility in directing prophylactic strategies. However, the methodological quality and applicability of existing models remain uncertain.

**Objective:**

This study aims to systematically identify and critically evaluate currently available risk prediction models for PMASD.

**Methods:**

PubMed, the Cochrane Library, Embase, Web of Science, CINAHL, Scopus, China National Knowledge Infrastructure (CNKI), China Science and Technology Journal Database (VIP), Wanfang Database, and Chinese Biomedical literature Database (CBM) were systematically searched from inception to 1 January 2026. Two researchers independently screened the literature and extracted and evaluated information based on the Prediction Model Risk of Bias Assessment Tool (PROBAST) and Data Extraction for Systematic Reviews of Prediction Modeling Studies (CHARMS). R4.4.0 software was used to conduct meta-analysis.

**Results:**

A total of 11 prediction models from 10 studies were included, with an incidence rate ranging from 22.8 to 59.1%. All studies indicated a substantial risk of bias, thus limiting their utility in clinical practice. The area under the curve (AUC) values of 11 models ranged from 0.812 to 0.914. The history of radiotherapy, type of stoma, stoma opening height, and surgical wound in the plate area were identified as the strongest predictors. In total, three studies validated the model externally, and six studies validated the model through an integration of internal and external methods, whereas one study did not undergo any validation after model development.

**Conclusion:**

In this systematic review, although most models performed well in terms of applicability, all models exhibited inherent limitations due to a high risk of bias. In the future, large-sample, multicenter, and high-quality prospective clinical studies should be carried out to optimize the predictive models, so as to improve their predictive ability and clinical application value.

**Systematic trial registration:**

identifier: CRD420251089071.

## 1 Introduction

Colorectal cancer is a leading cause of global cancer burden due to its elevated incidence and mortality, imposing a significant threat to human health. According to the global cancer statistics released by the International Agency for Research on Cancer (IARC) of the World Health Organization, colorectal cancer is the second most frequently identified malignancy in the world, and it ranks third in terms of fatalities from malignant diseases globally. In 2022, China reported more than 517,100 new colorectal cancer cases, accounting for 26.77% of the global total (1). It has become the third most commonly diagnosed cancer in terms of incidence and the fourth most frequent cause of cancer-related fatality in China (2). Enterostomy is a common and effective treatment for colorectal cancer, and alongside the growing prevalence of colorectal cancer, the number of patients requiring enterostomy is also steadily increasing (3). If patients receive inadequate care after surgery, a spectrum of stoma-related complications can arise, with Peristomal Moisture-Associated Skin Damage (PMASD) being the most predominant (4). In China, the reported rate of PMASD ranges from 31.2 to 59.1% (5, 6). PMASD is an inflammatory skin condition attributed to chronic contact with effluent and chemical irritants around the stoma (7, 8). Clinically, it manifests as erythema, ulceration, and may progress to infection and bleeding, accompanied by pain and itching (9), making it difficult for patients to properly wear ostomy pouches for effluent collection and triggering negative emotions such as anxiety and depression (10, 11). Furthermore, it escalates the difficulty of ostomy care management, increasing the frequency of follow-up visits and treatment costs for patients (12). Therefore, early identification and management of risk factors coupled with tailored interventions to prevent PMASD is clinically critical for patients.

Current PMASD assessment tools, such as the Studio Alterazoni Cutanee Stomale (13) and Peristomal Lesion Scale (14), are used to make a description and assessment of the area, location, and extent of erosion of the skin. However, they cannot identify those at high risk of PMASD. A Risk prediction model is a methodology that integrates multiple risk factors to forecast the probability and risk of specific events occurring, leveraging existing characteristic data (15). Currently, studies have constructed risk prediction models aiming at the early detection of patients at high risk of PMASD. These models incorporate diverse predictors, including demographic characteristics, clinical features, and psychosocial variables. Healthcare providers can leverage them to identify patients at high risk for PMASD early and implement timely preventive measures. However, the clinical implementation of these prediction models may be impeded by insufficient evidence regarding their predictive performance, risk of bias, and practical utility. At present, there is no systematic review to comprehensively evaluate risk prediction models for PMASD. Therefore, this study systematically reviews the performance and characteristics of current PMASD risk prediction models, which could offer evidence for the selection and further development of PMASD risk prediction models.

## 2 Methods

This systematic review was reported following the Preferred Reporting Items for Systematic Reviews and Meta-Analyses (PRISMA) (16).

### 2.1 Search strategy

A comprehensive search was conducted in PubMed, the Cochrane Library, Embase, Web of Science, CINAHL, Scopus, China National Knowledge Infrastructure (CNKI), China Science and Technology Journal Database (VIP), Wanfang Database, and Chinese Biomedical Literature Database (CBM) from inception to 1 January 2026. The search strategy combined the use of MeSH terms and free words, supplemented by manual searches to include references traced back. The following keywords were utilized for the search: “Enterostomy”, “Ostomy”, “Stoma”, “Colostomy”, “ileostomy”, “Fistulation”, “Dermatitis, Irritant”, “Dermatitides, Primary Irritant”, “contact dermatitis, irritant”, “dermatitis, irritative”, “nonallergic contact dermatitis”, “forecast model”, “predictive model”, “prediction model”, “clinical risk score”, “clinical prediction model”, “clinical scoring system”, and “risk assessment model”. A detailed search strategy is shown in the Supplementary material. Additionally, we manually retrieved reference lists of included studies to identify potential studies.

### 2.2 Inclusion and exclusion criteria

Inclusion criteria were as follows: (1) study design: cohort, case-control, and cross-sectional design; (2) population: patients with colostomy aged 18 years or above; (3) research content: the construction of a PMASD risk prediction model describing the establishment, verification, and evaluation process; (4) literature language: Chinese or English.

Exclusion criteria were as follows: (1) study protocols, reviews, conference abstracts, letters, and comments; (2) studies only analyzed the risk factors of PMASD without developing prediction models; (3) studies without a detailed process of developing models; (4) repeated publications; (5) the full text of the study is not available.

### 2.3 Study selection

Two reviewers independently screened studies according to the following procedures. First, duplicate studies were detected and removed. Second, titles and abstracts were screened to identify those that were eligible for inclusion in the study. Third, the full texts of the remaining studies were screened to identify eligible studies, and then the reference lists of included studies were examined to identify additional eligible studies. Any disagreement was resolved by consensus.

### 2.4 Data extraction

One reviewer performed data extraction using a predesigned form on the basis of the checklist for Critical Appraisal and Data Extraction for Systematic Reviews of Prediction Modelling Studies (CHARMS) (17), then another reviewer checked the information to ensure accuracy. Standardized tables were developed based on the factors and data extraction content of the prediction model, research bias risk, and suitability assessment. The data extraction content mainly includes the author, year of publication, study design, participants' characteristics, outcome, sample size, data source, incidence of events, number of candidate predictors, number of missing data and processing methods, predictors included in the model, model discrimination, degree of calibration, method of validation model, and model presentation form.

### 2.5 Risk of bias and application assessment

Two reviewers independently assessed the risk of bias and applicability of included models with the Prediction Model Risk of Bias Assessment Tool (PROBAST) checklist (18). This tool consists of four fields: participants, predictors, outcome, and analysis, and it employs 20 signaling questions to assess the risk of bias in the included studies. Each question is answered with “yes/maybe yes”, “probably not/no”, or “no information”. If the results of bias risk assessment in the four areas are all “low”, the overall risk of bias is judged to be “low”. If the assessment result of bias risk in ≥1 domains is “high”, the overall risk of bias is “high”. If the risk assessment of bias in one area is “unclear” and the risk assessment of bias in other areas is “low”, the overall risk of bias is considered “unclear”. In addition, for model development studies, even if all four domains were rated as low risk, it would still be high risk without external validation. However, model validation studies can still be considered as low risk as long as the construction of the verified model is based on a large dataset and internal verification is carried out during construction. PROBAST mainly evaluates the applicability in the first three fields, and the applicability evaluation method is similar to the bias risk evaluation method.

### 2.6 Statistical analysis

In this study, R 4.4.0 software was used to perform the meta-analysis. Heterogeneity was tested using the *I*^2^ test. If *I*^2^ < 50 %, this indicated no heterogeneity among the studies, and a fixed-effects model was used. If *I*^2^ ≥ 50 %, it indicated greater heterogeneity, and a random effects model was applied. Funnel plots and Egger's test were employed to identify publication bias. Symmetrical distribution of data points in the funnel plot and a *p*-value greater than 0.05 from Egger's test suggest no significant publication bias.

## 3 Results

### 3.1 Search results

**Figure 1:**
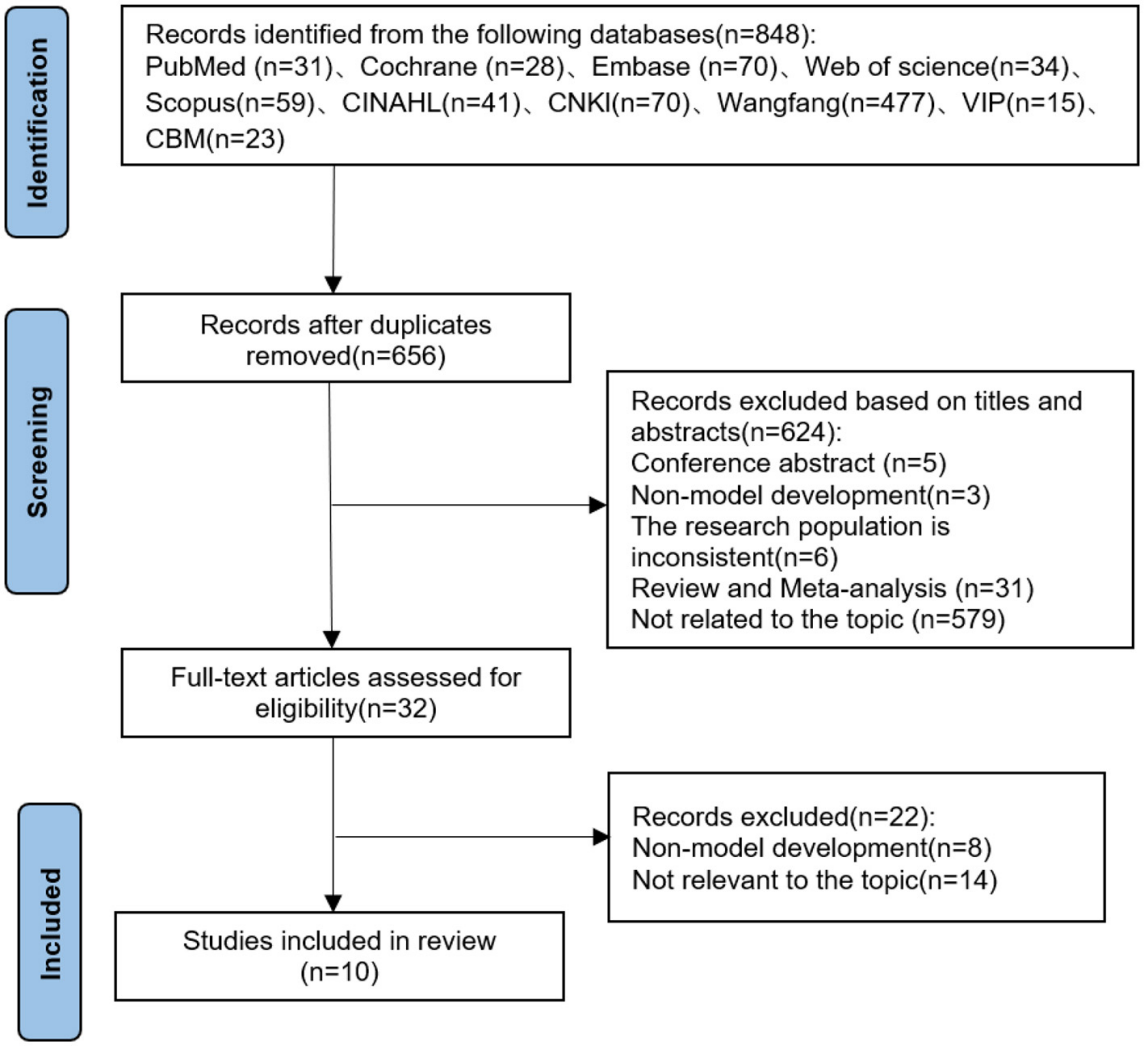
Flow diagram of study selection.

A total of 848 relevant studies were initially obtained, of which 192 were duplicates. 624 records were excluded after title and abstract screening. After a full-text review, 10 articles were ultimately included in the study (6, 19–27). The detailed process of study selection is shown in Figure 1.

### 3.2 Basic characteristics of included studies

These studies were published between 2021 and 2025. Among them, eight were published domestically (6, 20–22, 24–27), and two were published internationally (19, 23). In terms of study design, there were four retrospective studies (19, 21–23), two cross-sectional studies (6, 25) and three prospective studies (20, 24, 26). Seven studies (19–25) were single-center studies, and three studies (6, 26, 27) were multicenter studies. The estimated prevalence of PMASD ranged from 22.8 to 59.1%. The specific characteristics of the included studies are detailed in Table 1.

**Table 1:** Characteristics of included studies.

Study	Study design	Participants	Outcome	Sample size (model/validation, n)	Incidence of PMASD (%)	Data source
Chen et al. (19)	Retrospective cohort study	Older patients with enterostomy	Peristomal moisture-associated skin damage	162/68	42.59/41.18	Single-center study
Zhang et al. (20)	Prospective cohort study	Patients with colorectal cancer enterostomy	Peristomal moisture-associated skin damage	310/260	38.71/54.23	Single-center study
Zhang et al. (21)	Retrospective cohort study	Patients with enterostomy	Peristomal moisture-associated skin damage	220	34.09	Single-center study
Wu et al. (22)	Retrospective cohort study	Patients with enterostomy	Peristomal moisture-associated skin damage	268/101	56.5/34.0	Single-center study
Wang et al. (23)	Retrospective cohort study	Patients with colorectal cancer enterostomy	Peristomal moisture-associated skin damage	375/242	56.5/59.1	Single-center study
Jia etal. (24)	Prospective cohort study	Patients with enterostomy	Peristomal moisture-associated skin damage	337	22.8	Single-center study
Wei et al. (25)	Cross-sectional study	Patients with enterostomy	Fecal Dermatitis	516	34.1	Single-center study
Zhu et al. (6)	Cross-sectional study	Patients with enterostomy	Peristomal moisture-associated skin damage	292/94	31.2/35.1	Multi-center study
Liu et al. (26)	Prospective cohort study	Patients with colorectal cancer enterostomy	Peristomal moisture-associated skin damage	329/109	42.9/37.6	Multi-center study
Huang et al. (27)	Cross-sectional study	patients with colorectal cancer enterostomy	Peristomal moisture-associated skin damage	120	37.5	Multi-center study

### 3.3 Model development and predictors

A total of 11 prediction models were developed, encompassing a range of 9–28 candidate predictors. The top four important predictors were as follows: history of radiotherapy, type of stoma, stoma opening height, and surgical wound in the plate area. Regarding data missing and its processing methods, two studies did not specify whether any data were missing (6, 23), and three studies reported the reasons for missing data and directly excluded them (6, 19, 25). In the included studies, logistic regression and decision trees were utilized for model construction. The validation approaches differed across the studies: three employed external validation exclusively (22, 24, 27), whereas six others involved both internal and external validation (6, 19, 20, 23, 25, 26). Meanwhile, 1 model did not undergo any validation after development (21). Table 2 displays the basic characteristics of the included prediction models.

**Table 2:** Basic characteristics of risk prediction models for PMASD.

Study	Number of candidate predictors	Missing data	Missing data handling	Continuous variables	Modeling method	Selection of variables	Final predictors
Chen et al. (19)	11	45	Excluded	Remain unaltered	Logistic regression	Backward stepwise selection	Stoma height, surgical wound in plate area, ileostomy, history of radiotherapy, no preoperative stoma marking, peristomal skin folds, continuity of care
Zhang et al. (20)	12	None	—	All converted to categorical variables	Logistic regression	Lasso regression	Gender, surgical wound in plate area, plate attachment, soft diet, semi-fluid diet
Zhang et al. (21)	14	None	—	All converted to categorical variables	Logistic regression, decision tree	Univariate and multivariate analysis	Stoma leakage, history of radiotherapy or chemotherapy, self-care skill of stoma, stoma opening height, type of stoma
Wu et al. (24)	23	None	—	Remain unaltered	Logistic regression	Lasso regression	Gender, site of stoma, surgical wound in plate area, plate attachment, soft diet, semi-fluid diet
Wang et al. (23)	27	Not reported	Not reported	All converted to categorical variables	Logistic regression	Univariate and multivariate analysis	Fasting blood glucose, history of radiotherapy, stoma opening height, peristomal skin folds, defecation trait, type of stoma
Jia et al. (24)	28	None	—	All converted to categorical variables	Logistic regression	Univariate and multivariate analysis	Stoma leakage, time of using ostomy bag, diabetes, self-care skill of stoma
Wei et al. (25)	16	19	Excluded	Some converted to categorical variables	Logistic regression	Univariate and multivariate analysis	Family per capita monthly income, regular participation in ostomy fraternity, site of ostomy, periodic review of ostomy clinic, whether the bottom of ostomy bag was fitted, history of radiotherapy, whether anxiety, albumin level, malnutrition
Zhu et al. (6)	9	Not reported	Not reported	All converted to categorical variables	Logistic regression	Stepwise forward	Peristomal skin folds, stomy opening height, history of radiotherapy or chemotherapy, improper behavior in the use of stomy products, non-network continuous care model
Liu et al. (26)	20	45	Excluded	Remain unaltered	Logistic regression	Lasso regression	Gender, type of stoma, stoma height, surgical wound in plate area, plate attachment, soft diet, semi-fluid diet
Huang et al. (27)	13	None	—	All converted to categorical variables	Logistic regression	Univariate and multivariate analysis	Age, history of radiotherapy or chemotherapy, type of stoma, diabetes, stoma leakag, lack of knowledge of stoma care

### 3.4 Model performance and presentation

The overall area under the curve (AUC) values ranged from 0.812 to 0.914, indicating the discrimination was acceptable (28). The model calibration was reported in eight studies (6, 19, 20, 22–26), using either the Hosmer–Lemeshow test or calibration curve. Three studies employed decision curve analysis to verify the clinical utility of the prediction model (19, 25, 26). Four models were presented as model equations (20, 22, 26, 27), four models were presented as model equations and a nomogram (19, 23–25), one model was presented as a decision tree model (21), and one model was presented as both a nomogram and a scoring system in an app (6). The performances and presentations of prediction models were summarized in Table 3.

**Table 3:** Performance and presentation of risk prediction models for PMASD.

Study	Discrimination (AUC)	Calibration	Clinical effectiveness (DCA)	Model validation	Model presentation
Chen et al. (19)	A:0.90 (0.86–0.95) B:0.91 (0.84–0.98)	Hosmer–Lemeshow test, calibration curve	DCA	Internal + external	Model equation , nomogram
Zhang et al. (20)	A:0.858	Hosmer–Lemeshow test	—	Internal + external	Model equation
Zhang et al. (21)	Decision tree: 0.847 (0.794–0.900) logistic: 0.844 (0.791–0.897)	—	—	—	Nomogram, decision tree model
Wu et al. (22)	B:0.858	Hosmer–Lemeshow test	—	External	Model equation
Wang et al. (23)	A:0.820 (0.778–0.863) B:0.862 (0.815–0.909)	Hosmer–Lemeshow test	—	Internal + external	Model equation, nomogram
Jia et al. (24)	A:0.914 (0.873–0.955) B:0.905 (0.841–0.968)	Hosmer–Lemeshow test	—	External	Model equation , nomogram
Wei et al. (25)	A:0.895 (0.863–0.928) B:0.855 (0.792–0.918)	Hosmer–Lemeshow test, calibration curve	DCA	Internal + external	Model equation, Nomogram
Zhu et al. (6)	A:0.910 (0.872–0.948) B:0.841 (0.756–0.887)	Hosmer–Lemeshow test, calibration curve	DCA	Internal + external	Nomogram, scoring system
Liu et al. (26)	A:0.812 (0.765–0.858) B:0.860 (0.789–0.931)	Hosmer–Lemeshow test	—	Internal + external	Model equation
Huang et al. (27)	A:0.803 (0.684–0.872)	—	—	External	Model equation

A, development cohort; B, validation cohort.

### 3.5 Assessment of bias risk and applicability

The results of the risk of bias and applicability of included studies are summarized in Table 4.

**Table 4:** Evaluations of the bias risk and applicability of the included models.

Study	Risk of bias				Applicability			Overall	
	**Participants**	**Predictors**	**Outcome**	**Analysis**	**Participants**	**Predictors**	**Outcome**	**Risk of bias**	**Applicability**
Chen et al. (19)	H	L	L	H	L	L	L	H	L
Zhang et al. (20)	L	L	L	H	L	L	L	H	L
Zhang et al. (21)	H	L	L	H	L	L	L	H	L
Wu et al. (22)	H	L	L	H	L	L	L	H	L
Wang et al. (23)	H	U	L	H	L	L	U	H	U
Jia et al. (24)	L	L	L	H	L	L	U	H	U
Wei et al. (25)	H	L	L	H	L	L	L	H	L
Zhu et al. (6)	H	U	L	H	L	L	L	H	L
Liu et al. (26)	L	L	L	H	L	L	L	H	L
Huang et al. (27)	H	L	L	H	L	L	L	H	L

H, high risk of bias, high applicability risk; L, low risk of bias, low applicability risk; U, unclear.

Regarding the participant domain, the risk of bias was low in three studies (20, 24, 26) but high in the remaining seven (6, 19, 21–23, 25–27), which employed retrospective or cross-sectional designs.

In the predictor domain, two studies were deemed at unclear risk of bias (6, 23) due to insufficient reporting on quality control measures for predictor assessment.

In terms of the outcome domain, all studies were rated low. The definition of outcome variables adhered to established guidelines or recognized classification systems, and the timing between the assessment of predictors and outcomes was set based on clinical expertise.

Regarding the analysis domain, the overall 10 studies demonstrated a high risk of bias. A total of six studies (6, 22–24, 26, 27) were undermined by insufficient sample sizes; seven studies (6, 20, 21, 23–25, 27) transformed continuous variables into multi-categorical variables haphazardly; three studies (19, 25, 26) directly excluded missing data from the analysis; and two studies (6, 23) failed to report how it was handled. In terms of model performance evaluation, two studies (21, 27) did not report the calibration degree of the model, and five studies (20, 22–24, 26) evaluated calibration with only the Hosmer–Lemeshow test.

For the applicability concerns, eight studies included demonstrated good applicability across the participants, predictive factors, and outcomes aspects, therefore received a generally high applicability assessment (6, 19–22, 25–27). And for the remaining two studies (23, 24), the risk was deemed unclear due to insufficient details on the definition and assessment of outcome.

### 3.6 Meta-analysis results

**Figure 2:**
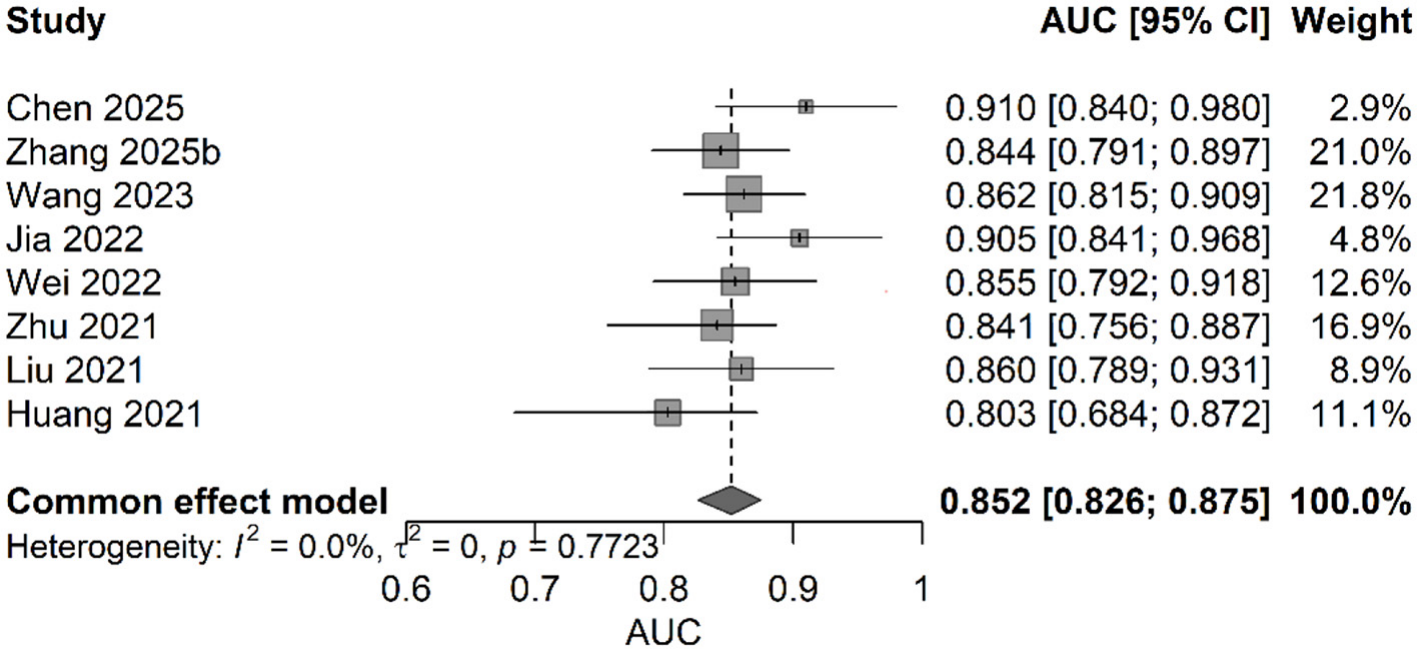
Forest plot of the pooled area under the curve (AUC) for the risk prediction model.

**Figure 3:**
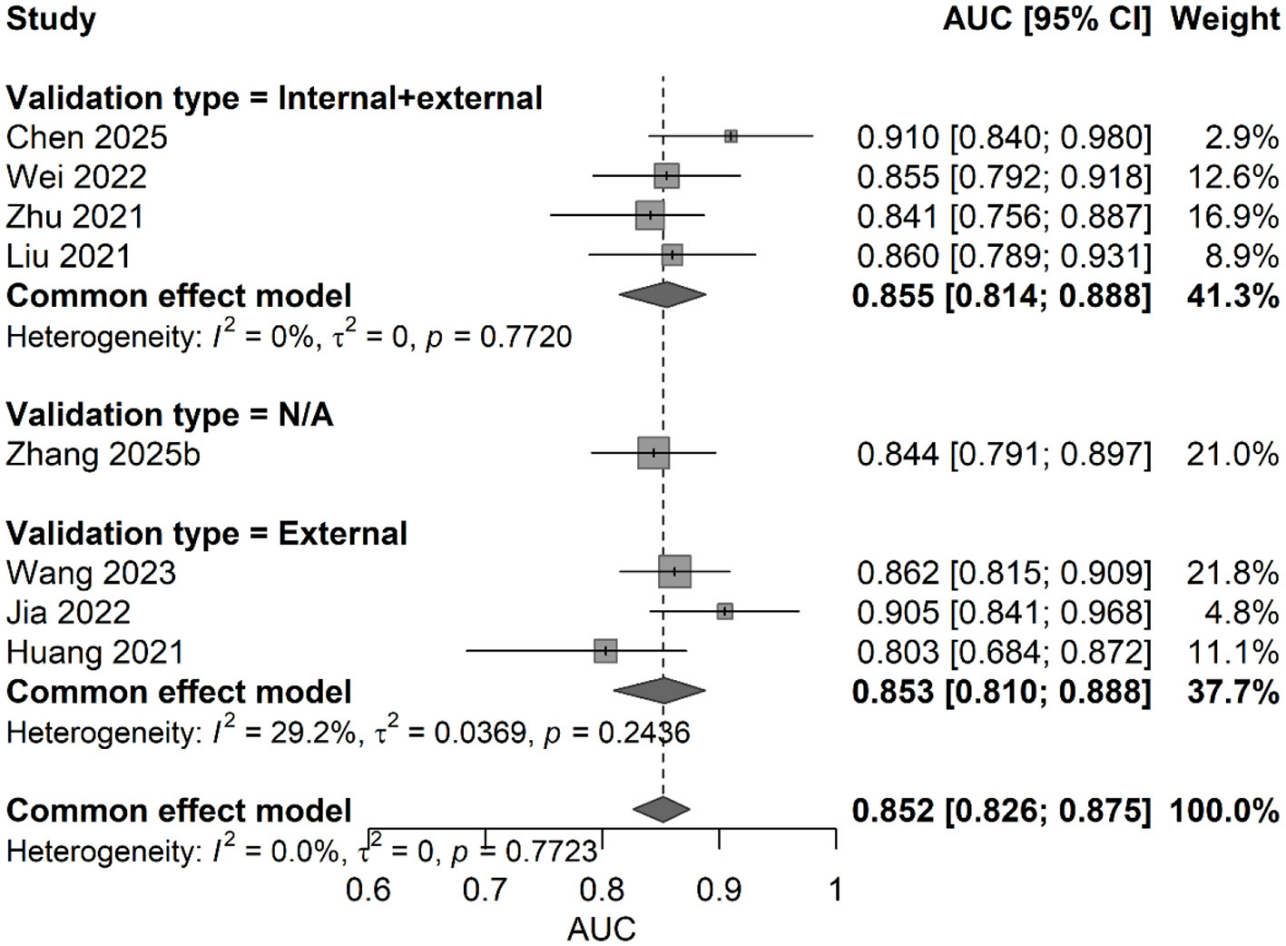
Forest plot of the subgroup analysis based on validation type for the pooled area under the curve (AUC).

**Figure 4:**
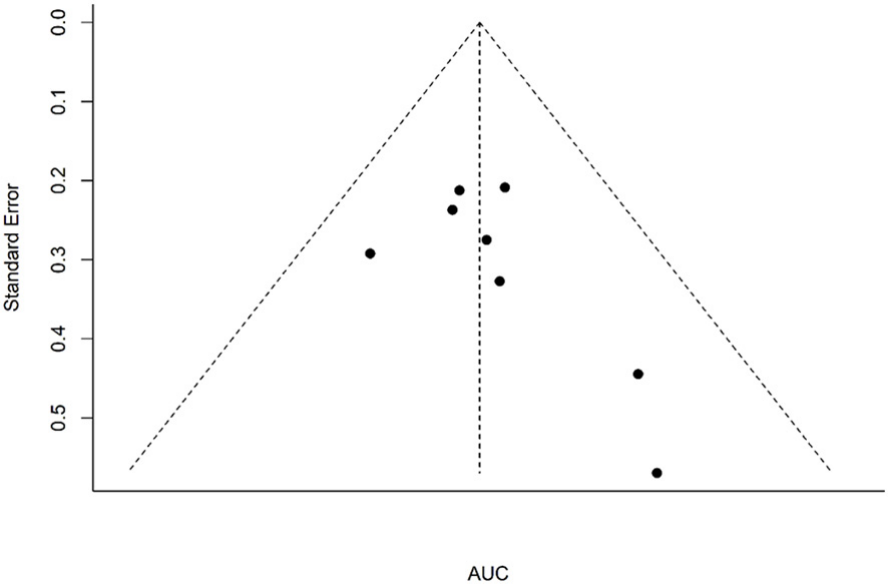
Funnel plot for risk of publication bias regarding the area under the curve (AUC).

Heterogeneity test indicated no significant heterogeneity among the included studies (*I*^2^ = 0.0%, *P* = 0.772), so a fixed-effects model was employed for the meta-analysis. In total, 8 of the 10 included studies reported the AUC values and their 95% confidence interval, with the respective AUC values being pooled in the meta-analysis. The meta-analysis yielded a pooled AUC value of 0.852 (95% confidence interval: 0.826–0.875), demonstrating good predictive discrimination. The specific forest plot is presented in Figure 2. A leave-one-out sensitivity analysis was conducted to assess the influence of individual studies on the overall effect size. The results demonstrated remarkable stability, with the recalculated pooled AUC ranging narrowly from 0.851 to 0.860 upon the systematic exclusion of each study. No single study was found to disproportionately drive the overall results, further confirming the robustness of the primary meta-analysis findings. Subgroup analysis stratified by validation type revealed consistently robust discriminative performance across all categories, with pooled AUC values ranging from 0.844 to 0.855 (Figure 3), indicating that the validation methodology did not significantly influence the models' overall predictive accuracy. Egger's test yielded a value of 1.710 (*p* = 0.138), suggesting no significant publication bias. The symmetrical appearance of the funnel plot and the even distribution of points around the true effect size suggest a high degree of consistency among the study findings. It provides further evidence against significant publication bias in this research. The funnel plot is shown in Figure 4.

## 4 Discussion

Early identification of high risk of PMASD is essential for preventing its occurrence and progression. This study presents a systematic review of current risk prediction models for PMASD in China. Finally, 11 models were developed based on the 10 studies that were included. We critically evaluated these models' characteristics, performance, risk of bias, and applicability, aiming to help select specific screening tools for high-risk PMASD patients. The earliest model was developed in 2021 (6, 26, 27), showing that scholarly attention to this problem has begun to gain momentum recently. All the included models showed good prediction performance, with the AUC values ranging from 0.812 to 0.914. However, all of them contained critical methodological issues in their statistical processing, thereby introducing a collectively high risk of bias. In total, two models (23, 24) were deemed to have an unclear applicability bias as a result of ambiguity in the outcome assessment, which may limit their practical utility. Overall, the models demonstrate promising predictive potential; nevertheless, this is accompanied by a substantial risk of bias. Future advances are required in the study design, the time interval between predictor and outcome assessment, the sample size, handling of missing values, and methods for predictor selection. Consequently, future studies must address the enhancement of current PMASD risk models. Furthermore, the development of new models through methodologically rigorous approaches is equally critical.

According to the PROBAST criteria (18), the high risk of bias primarily stemmed from key issues in the model development and validation. As for the study design, only three (20, 24, 26) of these studies are prospective cohort studies, which means that other studies neglected the blinding of the outcome determination and prediction information. Both predictors and outcomes require uniform definitions and measurement approaches throughout the study. These measurements need to be performed blindly at appropriate time points. Another notable issue is the inadequate sample size of the included studies. Seven of the studies (19–25) were single-center studies, and most studies included had the number of events per variable (EPV) < 20. Lower EPV values heighten the risk of the final model incorporating spurious predictors or omitting significant variables, and may result in model overfitting, compromising the accuracy of the models' extrapolated predictions (29). What is more, the inappropriate handling of missing data should be avoided. Three studies (19, 25, 26) directly excluded missing data from analysis, which may bias statistical analysis and reduce model reliability. Future studies are advised to employ multiple imputation or inverse probability weighting to appropriately address missing data and ensure full transparency in reporting both the processing procedures and their potential implications for research findings (30). Remarkably, seven studies arbitrarily transformed continuous variables into multi-categorical ones (6, 20, 21, 23–25, 27), thereby potentially adversely affecting the model's predictive performance due to a loss of information detail (31). To minimize misclassification bias, we recommend retaining continuous variables in their original form for statistical analyses and leveraging mathematical transformations or nonlinear regression for model fitting (32). With respect to model development methods, all included studies employed multivariate logistic regression, which facilitates the straightforward translation of models into clinically usable formats.

Regarding the clinical value of the models, besides risk of bias and applicability addressed in PROBAST, the incomplete reporting of model performance measures remains an issue that requires attention in current research. Model validation is a critical step in the predictive modeling process, from development to implementation. This process primarily encompasses both internal and external validation techniques (33). It is essential to externally validate a prediction model prior to its implementation. It determines whether the model's predictions remain accurate and generalizable across diverse populations and settings (34). Notably, all included studies were conducted exclusively in China, which significantly restricts the external validity and generalizability of the evaluated prediction models to other global populations. The risk factors and their predictive weights for PMASD can be heavily influenced by regional disparities, such as differences in healthcare systems, the availability of specialized stoma care resources, ethnic variations in skin characteristics, and differing dietary habits that alter stoma effluent composition. Consequently, the applicability of these models in non-Chinese demographic groups remains uncertain. Future studies should prioritize the external validation and potential recalibration of these prediction models across diverse international cohorts to ensure their global clinical utility. Internal validation is more straightforward to perform than external validation, as it involves assessing the model on the original development dataset (35). However, one study (21) only developed models without validation. Three of the included models did not conduct internal validation (22, 24, 27), which severely undermined the reliability and credibility of the predictive model development process, obscuring the models' true generalization ability and hindering effective model optimization, and ultimately increasing the risk and potential harm of model failure in practical applications. In short, implementing rigorous internal and external validation is a necessary cornerstone for building robust and generalizable predictive models. Eight studies (6, 19, 20, 22–26) conducted calibration tests for their models. For prediction models, calibration describes the degree of consistency between the predicted probabilities of a model and the actual observed event rates (36). Inconsistencies between the actual risk of disease and model-predicted probabilities may lead to resource waste and over-intervention (37). Besides, DCA was applied to three models (6, 19, 25), confirming their clinical usefulness. DCA estimates true-positive and false-positive rates based on the empirical distribution of risks (38). It provides greater clinical utility than the receiver operating characteristic (ROC) by quantifying the net benefit of prediction models across various threshold probabilities (38). In a word, it is imperative that future research defines effective strategies for clinical implementation and creates a unified methodology for the systematic assessment of prediction models.

The complexity of PMASD is driven by a mix of demographic and stoma-related risk factors. Due to this complexity, the selection of candidate predictors in all included studies was based on multi-dimensional factors. This review summarizes the most commonly reported factors. The top four factors identified were as follows: history of radiotherapy, type of stoma, stoma opening height, and surgical wound in the plate area. Radiotherapy can reduce the blood supply to the patient's mesenteric area and deteriorate their nutritional status. As a result, the patient's ability to resist adverse external factors decreases, making them more prone to skin damage (39). What's more, gastrointestinal reactions from radiotherapy, including diarrhea or constipation, can lead to stoma pouch leakage. This, in turn, increases the risk of PMASD by causing skin irritation and bag swelling (40). Patients with an ileostomy typically have a higher stool output than those with a colostomy, typically passing watery or loose stool that contains partially undigested digestive enzymes, making them more prone to peristomal skin damage (41). Additionally, most ileostomies are created as loop stomas, where the placement of a support rod complicates securement of the skin barrier. This often leads to over-enlarged aperture cutting in some patients, thereby increasing exposure of peristomal skin to fecal matter (42). The height of a stoma is the perpendicular distance measured from the highest point of the mucosa to the plane of the peristomal skin. When the height is insufficient, effluent fails to drain properly into the pouch and accumulates around the stomal orifice, which causes prolonged exposure of peristomal skin to effluent and results in PMASD (6). While current guidelines flag the ideal stoma height as 1–2 cm (43), Zhu's study (6) reveals that PMASD incidence increases significantly below 1.4 cm. Consequently, proactive preventive measures should be implemented when the stoma height is inappropriate. The wound and its exudate can lead to uneven skin around the stoma, which is easy to cause leakage, increasing the risk of wound infection and malignant leakage (44). This can delay wound healing, exacerbate basal plate leakage, and create a vicious cycle of ongoing peristomal skin damage. Hence, preoperative stoma siting is essential and should avoid the prior surgical incision. Moreover, for patients with a surgical wound near the stoma, the use of adjunct devices is recommended to enhance pouch security and mitigate leakage risk. Alternatively, hydrocolloid dressings can be applied to foster an optimal healing environment and accelerate wound closure (45). Given the complexity of PMASD, the accurate prediction of PMASD risk requires the integration of multiple variables, including gender, diet, and vital signs. Future studies need to ensure that well-documented risk factors, like those discussed above (type of stoma, height of stoma, plate attachment, and radiotherapy history), are accounted for.

While current PMASD prediction models demonstrate potential utility, their consistently high risk of bias necessitates cautious interpretation and application in clinical practice. To establish a more robust and comprehensive PMASD prediction model, future studies may benefit from prioritizing three key aspects: first, future models should prespecify candidate predictors according to clinical relevance and prior evidence, integrating universally recognized predictors with a broader spectrum of dynamic variables, such as peristomal skin environment and systemic inflammatory markers, to fully capture the multifaceted etiology of PMASD. Second, methodological rigor is imperative. Future model development should strictly utilize prospective cohort designs with adequate sample sizes to mitigate overfitting. Furthermore, statistical integrity should be maintained by preserving continuous variables in their original forms and employing multiple imputation strategies for missing data, rather than adopting simple exclusion or arbitrary dichotomization. Finally, transitioning these models from theoretical constructs to clinical applicability is crucial. Following rigorous internal and multicenter external validation, future predictive algorithms would be embedded into digital health applications or smart clinical assessment platforms. This technological integration will enable real-time, automated risk stratification, thereby facilitating personalized and proactive evidence-based stoma care.

## 5 Limitations

This systematic review has several potential limitations. First, the language of studies was confined to English and Chinese, which constitutes a potential source of bias. Second, the results were incompletely reported in some studies, precluding a meta-analysis of the predictive models' calibration. Third, despite our exhaustive literature search, it is possible that some gray literature was overlooked, thus potentially leading to an underestimation of the number of models. Fourth, all included studies were derived exclusively from Chinese cohorts. Consequently, the lack of ethnic and demographic diversity restricts the generalizability and transportability of these predictive models to other ethnic populations. Finally, all included studies were assessed as carrying a high risk of bias according to the PROBAST criteria. Key sources of bias primarily involve insufficient reporting of methodological details, inappropriate study design, and insufficient statistical rigor. The current limitations prevent us from endorsing any model for clinical application. Nevertheless, it establishes a foundation for future high-quality studies by demonstrating the need for rigorous methods and transparent reporting.

## 6 Conclusion

This systematic review identified 10 studies that developed 11 risk prediction models for PMASD in patients with enterostomy in China. While the high AUC values suggest promising statistical discrimination, these results must be interpreted with caution due to pervasive methodological limitations across all studies. Specifically, flaws such as the inadequate handling of missing data and insufficient sample sizes contribute to a universally high risk of bias that currently limits their clinical utility. In short, adherence to PROBAST standards is essential for future research, which ought to focus on validating existing models and creating rigorously developed new ones for PMASD prediction.

## Data Availability

The original contributions presented in the study are included in the article/Supplementary material, further inquiries can be directed to the corresponding author.
