# Supplementary material for: Risk prediction models for peristomal moisture-associated skin damage in China: a systematic review and meta-analysis
Source: Front Med (Lausanne). 2026 Jun 4;13:1789674. doi: 10.3389/fmed.2026.1789674 (PMC13275215; doi:10.3389/fmed.2026.1789674)
Supplement: Supplementary file 1 [file Supplementary_file_1.doc]

**检索策略**

**中文数据库**

**知网70**

（主题：肠造口术 + 肠造口 + 造口 + 造瘘 + 肠造瘘 + 人工肛门 + 结直肠癌造口+ 盲肠造口+ 回肠造口 + 结肠造口 + 十二直肠造口 + 空肠造口）AND（主题：潮湿相关性皮肤损伤 + 皮肤损伤 + 皮炎 + 潮湿相关性皮炎 + 粪水性皮炎 + 刺激性皮炎 + PMSD + 皮肤相关性并发症）AND（主题：风险 + 预测 + 风险预测 + 预测模型 + 风险预测模型 + 风险评估 + 危险因素 + 列线图 + 诺莫图 + 预测工具）

**万方477**

主题:(肠造口术 OR 肠造口 OR 造口 OR 造瘘 OR 肠造瘘 OR 人工肛门 OR 结直肠癌造口OR 盲肠造口OR 回肠造口 OR 结肠造口 OR 十二直肠造口 OR 空肠造口) and 主题:(潮湿相关性皮肤损伤 OR 皮肤损伤 OR 皮炎 OR潮湿相关性皮炎 OR 粪水性皮炎 OR 刺激性皮炎 OR PMSD OR 皮肤相关性并发症) and 主题:(风险 OR 预测 OR 风险预测 OR 预测模型 OR 风险预测模型 OR 风险评估 OR 危险因素 OR 列线图 OR 诺莫图 OR 预测工具)

**维普15**

题名或关键词:(肠造口术 OR 肠造口 OR 造口 OR 造瘘 OR 肠造瘘 OR 人工肛门 OR 结直肠癌造口OR 盲肠造口OR 回肠造口 OR 结肠造口 OR 十二直肠造口 OR 空肠造口) and 题名或关键词:(潮湿相关性皮肤损伤 OR 皮肤损伤 OR皮炎OR 潮湿相关性皮炎 OR 粪水性皮炎 OR 刺激性皮炎 OR PMSD OR 皮肤相关性并发症) and 题名或关键词:(风险 OR 预测 OR 风险预测 OR 预测模型 OR 风险预测模型 OR 风险评估 OR 危险因素 OR 列线图 OR 诺莫图 OR 预测工具)

**SinoMed23**

("肠造口术"[常用字段] OR "肠造口"[常用字段] OR "造口"[常用字段] OR "造瘘"[常用字段] OR "肠造瘘"[常用字段] OR "人工肛门"[常用字段] OR "结直肠癌造口OR 盲肠造口OR 回肠造口"[常用字段] OR "结肠造口"[常用字段] OR "十二直肠造口"[常用字段] OR "空肠造口"[常用字段]) AND( "潮湿相关性皮肤损伤"[常用字段] OR "皮肤损伤"[常用字段] OR "皮炎"[常用字段] OR "潮湿相关性皮炎"[常用字段] OR "粪水性皮炎"[常用字段] OR "刺激性皮炎"[常用字段] OR "PMSD"[常用字段] OR "皮肤相关性并发症"[常用字段]) AND( "风险"[常用字段] OR "预测"[常用字段] OR "风险预测"[常用字段] OR "预测模型"[常用字段] OR "风险预测模型"[常用字段] OR "风险评估"[常用字段] OR "危险因素"[常用字段] OR "列线图"[常用字段] OR "诺莫图"[常用字段] OR "预测工具"[常用字段])

**英文数据库**

**Pubmed**

| #1  20556 | Search: "Enterostomy"[Mesh] Sort by: Most Recent |
| --- | --- |
| #2  31628 | Search: (((((((((((((((enterostomy[Title/Abstract]) OR (Enterostomies[Title/Abstract])) OR (Ostomy[Title/Abstract])) OR (Ostomies[Title/Abstract])) OR (Stoma[Title/Abstract])) OR (Colostomy[Title/Abstract])) OR (Ileostomy[Title/Abstract])) OR (Intestinal Stoma[Title/Abstract])) OR (Fistulation[Title/Abstract])) OR (anus praeter[Title/Abstract])) OR (anus praeter naturalis[Title/Abstract])) OR (anus praeternaturalis[Title/Abstract])) OR (anus preternaturalis[Title/Abstract])) OR (artificial anus[Title/Abstract])) OR (mikulicz enterostomy[Title/Abstract])) OR (preternatural anus[Title/Abstract]) |
| #3  3620 | Search: "Dermatitis, Irritant"[Mesh] Sort by: Most Recent |
| #4  4713 | Search: ((((((((((((((((((((((((Dermatitis, Primary Irritant[Title/Abstract]) OR (Dermatitides, Primary Irritant[Title/Abstract])) OR (Irritant Dermatitides, Primary[Title/Abstract])) OR (Irritant Dermatitis, Primary[Title/Abstract])) OR (Primary Irritant Dermatitides[Title/Abstract])) OR (Primary Irritant Dermatitis[Title/Abstract])) OR (Irritant Dermatitis[Title/Abstract])) OR (Dermatitides, Irritant[Title/Abstract])) OR (Irritant Dermatitides[Title/Abstract])) OR (contact dermatitis, irritant[Title/Abstract])) OR (dermatitis contacta irritativa[Title/Abstract])) OR (dermatitis irritativa[Title/Abstract])) OR (dermatitis, irritative[Title/Abstract])) OR (irritative contact dermatitis[Title/Abstract])) OR (irritative dermatitis[Title/Abstract])) OR (non-allergic contact dermatitis[Title/Abstract])) OR (nonallergic contact dermatitis[Title/Abstract])) OR (irritant contact dermatitis[Title/Abstract])) OR (moisture-associated skin damage[Title/Abstract])) OR (moisture-related skin damage[Title/Abstract])) OR (moisture-associated skin injury[Title/Abstract])) OR (moisture-related skin injury[Title/Abstract])) OR (MASD[Title/Abstract])) OR (Fecal dermatitis[Title/Abstract]) OR (Skin-related complication[Title/Abstract])) OR (moisture-related dermatitis[Title/Abstract]) OR (moisture- associated dermatitis[Title/Abstract]) |
| #5  3893097 | Search: (((((((((((((((((forecast model[Title/Abstract]) OR (forecast modeling[Title/Abstract])) OR (forecast modelling[Title/Abstract])) OR (forecast simulation[Title/Abstract])) OR (forecasting model[Title/Abstract])) OR (predictive modeling[Title/Abstract])) OR (predictive modelling[Title/Abstract])) OR (predictive simulation[Title/Abstract])) OR (predictive model[Title/Abstract])) OR (prediction model[Title/Abstract])) OR (clinical risk score[Title/Abstract])) OR (clinical prediction model[Title/Abstract])) OR (clinical predictive model[Title/Abstract])) OR (clinical scoring system[Title/Abstract])) OR (risk assessment model[Title/Abstract])) OR (risk prediction model[Title/Abstract])) OR (risk predictive model[Title/Abstract])) OR (prediction[Title/Abstract] OR predictor[Title/Abstract]) OR (risk[Title/Abstract]) OR (risk assessment[Title/Abstract]) OR (nomogram[Title/Abstract]) OR (Prediction tool[Title/Abstract]) OR (assessment tool[Title/Abstract]) |
| #6  41444 | Search: ("Enterostomy"[Mesh]) OR ((((((((((((((((enterostomy[Title/Abstract]) OR (Enterostomies[Title/Abstract])) OR (Ostomy[Title/Abstract])) OR (Ostomies[Title/Abstract])) OR (Stoma[Title/Abstract])) OR (Colostomy[Title/Abstract])) OR (Ileostomy[Title/Abstract])) OR (Intestinal Stoma[Title/Abstract])) OR (Fistulation[Title/Abstract])) OR (anus praeter[Title/Abstract])) OR (anus praeter naturalis[Title/Abstract])) OR (anus praeternaturalis[Title/Abstract])) OR (anus preternaturalis[Title/Abstract])) OR (artificial anus[Title/Abstract])) OR (mikulicz enterostomy[Title/Abstract])) OR (preternatural anus[Title/Abstract])) |
| #7  7200 | Search: ("Dermatitis, Irritant"[Mesh]) OR (((((((((((((((((((((((((Dermatitis, Primary Irritant[Title/Abstract]) OR (Dermatitides, Primary Irritant[Title/Abstract])) OR (Irritant Dermatitides, Primary[Title/Abstract])) OR (Irritant Dermatitis, Primary[Title/Abstract])) OR (Primary Irritant Dermatitides[Title/Abstract])) OR (Primary Irritant Dermatitis[Title/Abstract])) OR (Irritant Dermatitis[Title/Abstract])) OR (Dermatitides, Irritant[Title/Abstract])) OR (Irritant Dermatitides[Title/Abstract])) OR (contact dermatitis, irritant[Title/Abstract])) OR (dermatitis contacta irritativa[Title/Abstract])) OR (dermatitis irritativa[Title/Abstract])) OR (dermatitis, irritative[Title/Abstract])) OR (irritative contact dermatitis[Title/Abstract])) OR (irritative dermatitis[Title/Abstract])) OR (non-allergic contact dermatitis[Title/Abstract])) OR (nonallergic contact dermatitis[Title/Abstract])) OR (irritant contact dermatitis[Title/Abstract])) OR (moisture-associated skin damage[Title/Abstract])) OR (moisture-related skin damage[Title/Abstract])) OR (moisture-associated skin injury[Title/Abstract])) OR (moisture-related skin injury[Title/Abstract])) OR (MASD[Title/Abstract])) OR (Fecal dermatitis[Title/Abstract]) OR (Skin-related complication[Title/Abstract])) OR (moisture-related dermatitis[Title/Abstract]) OR (moisture- associated dermatitis[Title/Abstract])) |
| #8  31 | #5 AND #6 AND #7 |

Web of Science

| #1  39539 | (TS=(enterostomy OR Enterostomies OR Ostomy OR Ostomies OR Stoma OR Colostomy OR Ileostomy OR Intestinal Stoma OR Fistulation OR anus praeger OR anus praeger naturalis OR anus praeternaturalis OR anus praeternaturalis OR artificial anus OR mikulicz enterostomy OR preternatural anus)) |
| --- | --- |
| #2  5042 | (TS=(Dermatitis, Primary Irritant OR Dermatitides, Primary Irritant OR Irritant Dermatitides, Primary OR Irritant Dermatitis, Primary OR Primary Irritant Dermatitides OR Primary Irritant Dermatitis OR Irritant Dermatitis OR Dermatitides, Irritant OR Irritant Dermatitides OR contact dermatitis, irritant OR dermatitis contacta irritativa OR dermatitis irritativa OR dermatitis, irritative OR irritative contact dermatitis OR irritative dermatitis OR non-allergic contact dermatitis OR nonallergic contact dermatitis OR irritant contact dermatitis OR moisture-associated skin damage OR moisture-related skin damage OR moisture-associated skin injury OR moisture-related skin injury OR MASD OR Fecal dermatitis OR Skin-related complication OR moisture-related dermatitis OR moisture- associated dermatitis)) |
| #3  8111630 | (TS=(forecast model OR forecast modeling OR forecast modelling OR forecast simulation OR forecasting model OR predictive modeling OR predictive modelling OR predictive simulation OR predictive model OR prediction model OR clinical risk score OR clinical prediction model OR clinical predictive model OR clinical scoring system OR risk assessment model OR risk prediction model OR risk predictive model OR prediction OR predictor OR risk OR risk assessment OR nomogram OR Prediction tool OR assessment tool)) |
| #4  34 | #1 AND #2 AND #3 |

Embase

| #1  51670 | 'enterostomy'/exp |
| --- | --- |
| #2  80562 | 'enterostomy' OR 'enterostomies' OR 'ostomy' OR 'ostomies' OR 'stoma' OR 'colostomy' OR 'ileostomy' OR 'intestinal stoma' OR 'fistulation' OR 'anus praeger' OR 'anus praeger naturalis' OR 'anus praeternaturalis' OR 'artificial anus' OR 'mikulicz enterostomy' OR 'preternatural anus' |
| #3  5615 | 'irritant contact dermatitis'/exp |
| #4  5088 | 'dermatitis, primary irritant' OR 'dermatitides, primary irritant' OR 'irritant dermatitides, primary' OR 'irritant dermatitis, primary' OR 'primary irritant dermatitides' OR 'primary irritant dermatitis' OR 'irritant dermatitis' OR 'irritant dermatitis' OR 'dermatitides, irritant' OR 'irritant dermatitides' OR 'contact dermatitis, irritant' OR 'contact dermatitis, irritant' OR 'dermatitis contacta irritativa' OR 'dermatitis contacta irritativa' OR 'dermatitis irritativa' OR 'dermatitis irritativa' OR 'dermatitis, irritative' OR 'dermatitis, irritative' OR 'irritative contact dermatitis' OR 'irritative contact dermatitis' OR 'irritative dermatitis' OR 'irritative dermatitis' OR 'non-allergic contact dermatitis' OR 'non-allergic contact dermatitis' OR 'nonallergic contact dermatitis' OR 'nonallergic contact dermatitis' OR 'irritant contact dermatitis' OR 'irritant contact dermatitis' OR 'dermatitis,irritant' OR 'dermatitis,irritant' OR 'moisture-related skin damage' OR 'moisture-related skin jnjury' OR 'moisture-associated skin damage' OR 'fecal dermatitis' OR 'masd' OR 'moisture-associated skin injury' OR 'Skin-related complication' OR 'moisture-related dermatitis' OR 'moisture- associated dermatitis' |
| #5  44709 | 'predictive model'/exp |
| #6  6997481 | 'forecast model' OR 'forecast modeling' OR 'forecast modelling' OR 'forecast simulation' OR 'forecasting model' OR 'predictive modeling' OR 'predictive modelling' OR 'predictive simulation' OR 'predictive model' OR 'prediction model' OR 'clinical risk score' OR 'clinical prediction model' OR 'clinical predictive model' OR 'clinical scoring system' OR 'risk assessment model' OR 'risk prediction model' OR 'risk predictive model' OR 'prediction' OR 'predictor' OR 'risk' OR 'risk assessment' OR 'nomogram' OR 'prediction tool' OR 'assessment tool' |
| #7  89260 | #1 OR #2 |
| #8  7211 | #3 OR #4 |
| #9  6997508 | #5 OR #6 |
| #10  70 | #7 AND #8 AND #9 |

Cochrane

| #1  659 | MeSH descriptor: [Enterostomy] explode all trees |
| --- | --- |
| #2  3853 | (enterostomy OR Enterostomies OR Ostomy OR Ostomies OR Stoma OR Colostomy OR Ileostomy OR Intestinal Stoma OR Fistulation OR anus praeger OR anus praeger naturalis OR anus praeternaturalis OR anus praeternaturalis OR artificial anus OR mikulicz enterostomy OR preternatural anus) |
| #3  329 | MeSH descriptor: [Dermatitis, Irritant] explode all trees |
| #4  1356 | (dermatitis, primary irritant OR dermatitides, primary irritant OR irritant dermatitides, primary OR irritant dermatitis, primary OR primary irritant dermatitides OR primary irritant dermatitis OR irritant dermatitis OR irritant dermatitis OR dermatitides, irritant OR irritant dermatitides OR contact dermatitis, irritant OR contact dermatitis, irritant OR dermatitis contacta irritativa OR dermatitis contacta irritativa OR dermatitis irritativa OR dermatitis irritativa OR dermatitis, irritative OR dermatitis, irritative OR irritative contact dermatitis OR irritative contact dermatitis OR irritative dermatitis OR irritative dermatitis OR non-allergic contact dermatitis OR non-allergic contact dermatitis OR nonallergic contact dermatitis OR nonallergic contact dermatitis OR irritant contact dermatitis OR irritant contact dermatitis OR dermatitis, irritant OR dermatitis, irritant OR moisture-related skin damage OR moisture-related skin jnjury OR moisture-associated skin damage OR fecal dermatitis OR masd OR moisture-associated skin injury OR Skin-related complication OR moisture-related dermatitis OR moisture-associated dermatitis) |
| #5  1031221 | (forecast model OR forecast modeling OR forecast modelling OR forecast simulation OR forecasting model OR predictive modeling OR predictive modelling OR predictive simulation OR predictive model OR prediction model OR clinical risk score OR clinical prediction model OR clinical predictive model OR clinical scoring system OR risk assessment model OR risk prediction model OR risk predictive model OR prediction OR predictor OR risk OR risk assessment OR nomogram OR Prediction tool OR assessment tool) |
| #6  3957 | #1 OR #2 |
| #7  1454 | #3 OR #4 |
| #8  28 | #5 AND #6 AND #7 |

Scopus

| #1  80768 | TITLE-ABS-KEY ( "enterostomy" OR "Enterostomies" OR "Ostomy" OR "Ostomies" OR "Stoma" OR "Colostomy" OR "ileostomy" OR "Intestinal Stoma" OR "Fistulation" OR "anus praeter" OR "anus praeter naturalis" OR "anus praeternaturalis" OR "anus preternaturalis" OR "artificial anus" OR "mikulicz enterostomy" OR "preternatural anus" ) |
| --- | --- |
| #2  5563 | TITLE-ABS-KEY ( "dermatitis, primary irritant" OR "dermatitides, primary irritant" OR "irritant dermatitides, primary" OR "irritant dermatitis, primary" OR "primary irritant dermatitides" OR "primary irritant dermatitis" OR "irritant dermatitis" OR "irritant dermatitis" OR "dermatitides, irritant" OR "irritant dermatitides" OR "contact dermatitis, irritant" OR "contact dermatitis, irritant" OR "dermatitis contacta irritativa" OR "dermatitis contacta irritativa" OR "dermatitis irritativa" OR "dermatitis irritativa" OR "dermatitis, irritative" OR "dermatitis, irritative" OR "irritative contact dermatitis" OR "irritative contact dermatitis" OR "irritative dermatitis" OR "irritative dermatitis" OR "non-allergic contact dermatitis" OR "non-allergic contact dermatitis" OR "nonallergic contact dermatitis" OR "nonallergic contact dermatitis" OR "irritant contact dermatitis" OR "irritant contact dermatitis" OR "dermatitis,irritant" OR "dermatitis,irritant" OR "moisture-related skin damage" OR "moisture-related skin jnjury" OR "moisture-associated skin damage" OR "moisture-associated skin injury" OR "fecal dermatitis" OR "masd" OR "Skin-related complication" OR "moisture-related dermatitis" OR "moisture- associated dermatitis" ) |
| #3  9536850 | TITLE-ABS-KEY ( "forecast model" OR "forecast modeling" OR "forecast modelling" OR "forecast simulation" OR "forecasting model" OR "predictive modeling" OR "predictive modelling" OR "predictive simulation" OR "predictive model" OR "prediction model" OR "clinical risk score" OR "clinical prediction model" OR "clinical predictive model" OR "clinical scoring system" OR "risk assessment model" OR "risk prediction model" OR "risk predictive model" OR "prediction" OR "predictor" OR "risk" OR "risk assessment" OR "nomogram" OR "prediction tool" OR "assessment tool" ) |
| #4  59 | #1 AND #2 AND #3 |

CINAHL

| 41 | ((enterostomy OR Enterostomies OR Ostomy OR Ostomies OR Stoma OR Colostomy OR Ileostomy OR Intestinal Stoma OR Fistulation OR anus praeger OR anus praeger naturalis OR anus praeternaturalis OR anus praeternaturalis OR artificial anus OR mikulicz enterostomy OR preternatural anus)) AND ((Dermatitis, Primary Irritant OR Dermatitides, Primary Irritant OR Irritant Dermatitides, Primary OR Irritant Dermatitis, Primary OR Primary Irritant Dermatitides OR Primary Irritant Dermatitis OR Irritant Dermatitis OR Dermatitides, Irritant OR Irritant Dermatitides OR contact dermatitis, irritant OR dermatitis contacta irritativa OR dermatitis irritativa OR dermatitis, irritative OR irritative contact dermatitis OR irritative dermatitis OR non-allergic contact dermatitis OR nonallergic contact dermatitis OR irritant contact dermatitis OR moisture-associated skin damage OR moisture-related skin damage OR moisture-associated skin injury OR moisture-related skin injury OR MASD OR Fecal dermatitis OR Skin-related complication OR moisture-related dermatitis OR moisture- associated dermatitis)) AND ((forecast model OR forecast modeling OR forecast modelling OR forecast simulation OR forecasting model OR predictive modeling OR predictive modelling OR predictive simulation OR predictive model OR prediction model OR clinical risk score OR clinical prediction model OR clinical predictive model OR clinical scoring system OR risk assessment model OR risk prediction model OR risk predictive model OR prediction OR predictor OR risk OR risk assessment OR nomogram OR Prediction tool OR assessment tool)) |
| --- | --- |
